# Preexisting maternal immunity to AAV but not Cas9 impairs in utero gene editing in mice

**DOI:** 10.1172/JCI179848

**Published:** 2024-05-09

**Authors:** John S. Riley, Valerie L. Luks, Cara L. Berkowitz, Ana Maria Dumitru, Nicole J. Kus, Apeksha Dave, Pallavi Menon, Monique E. De Paepe, Rajan Jain, Li Li, Lorraine Dugoff, Christina Paidas Teefey, Mohamad-Gabriel Alameh, Philip W. Zoltick, William H. Peranteau

**Affiliations:** 1 Center for Fetal Research, Children’s Hospital of Philadelphia, Philadelphia, Pennsylvania, USA.; 2 Department of Surgery, Hospital of the University of Pennsylvania, Philadelphia, Pennsylvania, USA.; 3 Department of Pathology and Laboratory Medicine, Brown University, Providence, Rhode Island, USA.; 4 Division of Cardiology, Department of Medicine, and; 5 Division of Maternal-Fetal Medicine, Department of Obstetrics and Gynecology, Hospital of the University of Pennsylvania, Philadelphia, Pennsylvania, USA.; 6 Center for Fetal Diagnosis and Treatment and; 7 Department of Pathology and Laboratory Medicine, Children’s Hospital of Philadelphia, Philadelphia, Pennsylvania, USA.

**Keywords:** Immunology, Therapeutics, Gene therapy, Genetic diseases

## Abstract

In utero gene editing (IUGE) is a potential treatment for inherited diseases that cause pathology before or soon after birth. Preexisting immunity to adeno-associated virus (AAV) vectors and Cas9 endonuclease may limit postnatal gene editing. The tolerogenic fetal immune system minimizes a fetal immune barrier to IUGE. However, the ability of maternal immunity to limit fetal gene editing remains a question. We investigated whether preexisting maternal immunity to AAV or Cas9 impairs IUGE. Using a combination of fluorescent reporter mice and a murine model of a metabolic liver disease, we demonstrated that maternal anti-AAV IgG antibodies were efficiently transferred from dam to fetus and impaired IUGE in a maternal titer–dependent fashion. By contrast, maternal cellular immunity was inefficiently transferred to the fetus, and neither maternal cellular nor humoral immunity to Cas9 impaired IUGE. Using human umbilical cord and maternal blood samples collected from mid- to late-gestation pregnancies, we demonstrated that maternal-fetal transmission of anti-AAV IgG was inefficient in midgestation compared with term, suggesting that the maternal immune barrier to clinical IUGE would be less relevant at midgestation. These findings support immunologic advantages for IUGE and inform maternal preprocedural testing protocols and exclusion criteria for future clinical trials.

## Abstract 2

Maternal immunity against the adeno-associated virus but not Cas9 can limit therapeutic prenatal gene editing when used to deliver the gene-editing technology.

## Graphical abstract

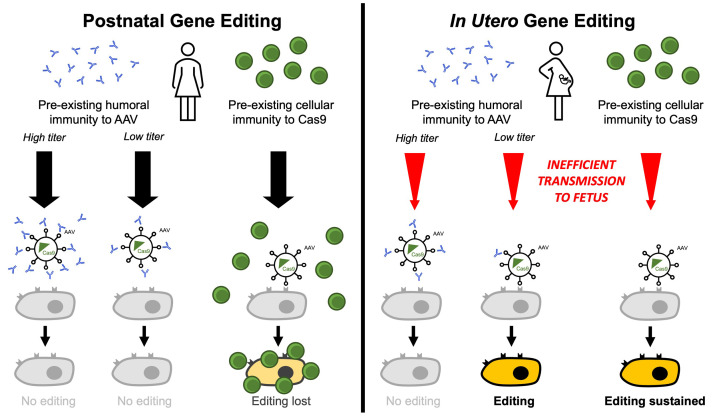

## Introduction

In utero gene editing (IUGE) is a potential treatment for inherited diseases that takes advantage of normal fetal developmental properties to institute the therapy before the onset of pathology. It has the potential to provide a one-and-done treatment for several diseases that cause pathology in early childhood, including lysosomal storage diseases, urea cycle disorders, and other metabolic liver diseases. We previously demonstrated the ability of in utero base editing to correct the disease phenotype in the mouse models of hereditary tyrosinemia type 1 (HT1) (1) and mucopolysaccharidosis type I (MPS I, Hurler syndrome) (2). These studies illustrated several potential advantages of a fetal gene editing strategy, including the immaturity of the fetal immune response to non-self antigens. Specifically, these studies used either adeno-associated virus (AAV) or adenovirus to deliver a cytosine or adenine base editor transgene to fetal or adult recipients. Humoral immune responses to the viral vectors and *Streptococcus pyogenes* Cas9 endonuclease (SpCas9), the backbone of the base editors, were observed following adult but not fetal gene editing.

While the absence of a de novo fetal immune response following IUGE is indeed an advantage in comparison with a postnatal gene editing strategy, preexisting immunity to the naturally derived components of gene editing technology must also be considered in the context of IUGE. It has long been recognized that preexisting immunity to AAV as a result of previous infection is common, present in 7.7%–15% of infants (3, 4), 21.5%–54% of children/young adults (4, 5), and 20%–60% of adults (6, 7) varying by serotype. When present at sufficient titer, these antibodies impair successful vector binding to target cells, negating efficacy in both preclinical animal models and clinical trials (8–11). More recently, preexisting immunity to bacteria-derived Cas9 orthologs has also been recognized as common among adults (SpCas9, 58%; *Staphylococcus aureus* Cas9 [SaCas9], 78%) (12). In this case, cytotoxic T cells are the critical component of the immune response, having been shown in preclinical animal studies to destroy successfully edited hepatocytes and myocytes as a result of neoantigen presentation in the context of MHC class I (13, 14).

Direct fetal infection by bacteria or viruses present in the external environment is uncommon in normal pregnancy because of protection by the amniotic sac and cervix (15). While vertical transmission of pathogens from the mother to the fetus does occur, only select pathogens (e.g., TORCH pathogens, Zika virus) are able to circumvent the innate immune defenses of the syncytiotrophoblasts and extravillous trophoblasts that form the fetal interface of the placenta (16). We would therefore not expect the fetus itself to have preexisting immunity to the naturally derived components of gene editing technology. However, the interconnectedness of the maternal and fetal immune systems is well established. Vertical transmission of IgG antibodies during pregnancy provides critical immune protection to common pathogens in the neonatal period (17–21) and is the probable source of the aforementioned preexisting anti-AAV IgG antibodies detected in infants (3, 4). Maternal-fetal T cell trafficking in normal pregnancy is also described and is an important mechanism for reciprocal tolerance at the maternal-fetal interface (22, 23). We and others have demonstrated that this interplay between the maternal and fetal immune systems can have important effects on and/or be altered by fetal cellular therapies, including rapid rejection of third-party fetal allotransplants by preexisting or induced maternal donor-specific antibodies (24, 25) and increased maternal-fetal T cell trafficking following fetal injection (26, 27). Notably, fostering injected fetuses immediately after birth can mitigate the negative impact of induced maternal antibodies transferred in breast milk but may have no effect on a preexisting maternal immune barrier. As the potential therapeutic benefits of IUGE become more apparent, it is necessary to consider the potential limitations posed by preexisting maternal immunity to the naturally derived components of gene editing technology. Investigation of these potential limitations is a critical step for advancing this field toward successful clinical translation.

In the current study, we investigated the effect of preexisting maternal immunity to AAV and Cas9 on the efficiency of IUGE in a fluorescent reporter mouse model with low baseline liver editing and a mouse model of HT1 in which high liver editing can be achieved. We showed that preexisting maternal anti-AAV immunity but not anti-Cas9 immunity can impair fetal editing. Furthermore, we performed translational studies to assess vertical antibody transmission efficiency using sera collected from human maternal-fetal/neonatal dyads across a range of gestational ages and observed inefficient anti-AAV antibody transmission from the mother to the fetus before the third trimester. These studies demonstrate immunologic advantages for IUGE and will inform maternal preprocedural testing protocols and exclusion criteria for future clinical trials.

## Results

### Maternal anti-AAV IgG is efficiently transferred to the murine fetus before birth.

**Figure 1:**
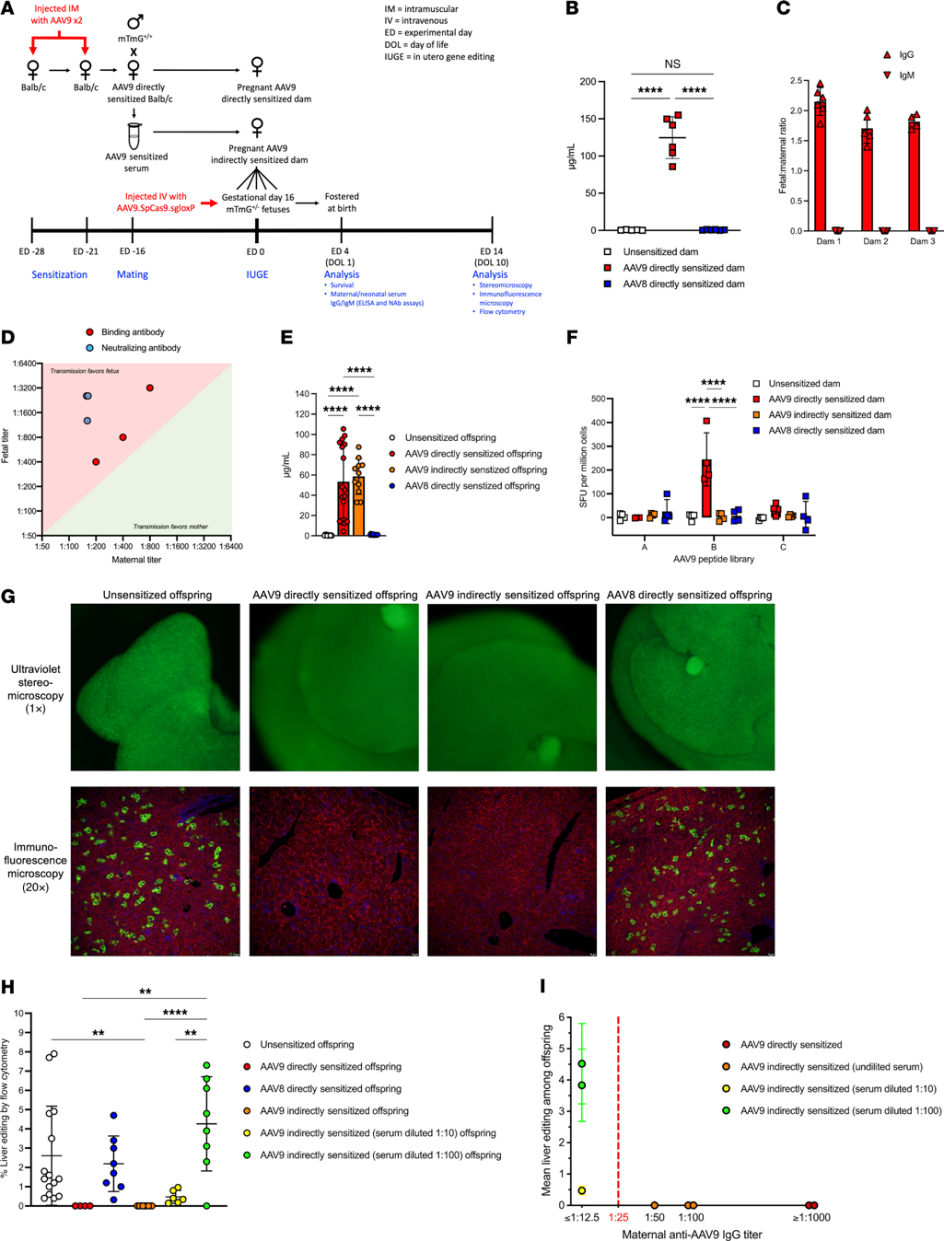
Preexisting maternal humoral immunity to AAV impairs fetal gene editing. (**A**) Experimental design. (**B**) Anti-AAV9 IgG binding antibody (BAb). BAb concentration was assessed by ELISA and compared among the groups using 1-way ANOVA. (**C**) Maternal-fetal transmission of anti-AAV9 antibodies. At delivery, serum was collected from dams and newborn fetuses, and the concentration of anti-AAV9 IgG and IgM was compared to generate a fetal/maternal ratio. (**D**) Comparison of maternal and fetal anti-AAV9 BAb and neutralizing antibody (NAb) titer. Notably, neonatal blood collection is prone to hemolysis, which interferes with luciferase-based assays such as NAb titration. (**E**) Fetal antibody concentration by group. Anti-AAV9 IgG BAb concentrations were assessed in the serum of uninjected offspring at birth and compared among the groups using 1-way ANOVA. (**F**) Anti-AAV9 IFN-γ enzyme-linked immunosorbent spot (ELISPOT). Spot-forming units (SFU) per million cells were compared for each AAV9 peptide library by 2-way ANOVA. (**G**) Ultraviolet stereomicroscopy and immunofluorescence microscopy. Livers of unsensitized offspring and AAV8 directly sensitized offspring showed numerous bright green (mG^+^) cells by ultraviolet stereomicroscopy and immunofluorescence microscopy, confirming successful gene editing. By contrast, livers of AA9 directly and indirectly sensitized offspring showed no mG^+^ cells, indicating impaired gene editing. (**H**) Quantification of fetal liver gene editing by flow cytometry. Shown is percent mG^+^mT^–^/(mG^+^mT^–^ + mG^–^mT^+^) among live CD45^–^TER119^–^CD31^–^EpCAM^–^E-cadherin^+^ offspring hepatocytes. Mean liver editing was statistically equal among unsensitized offspring, AAV8 directly sensitized offspring, and AAV9 indirectly sensitized (serum diluted 1:100) offspring by 1-way ANOVA. Low-level editing was observed among AAV9 indirectly sensitized (1:10) offspring, and no editing was observed among AAV9 directly and indirectly sensitized (undiluted serum) offspring. (**I**) Fetal liver editing correlated with maternal anti-AAV9 IgG titer. Fetal gene editing was absent when maternal titer was greater than 1:25 and restored when it was less than 1:25. **P < 0.01, ****P < 0.0001.

We first assessed AAV immunity following maternal sensitization and the efficiency of maternal-fetal transmission of anti-AAV antibodies in murine pregnancy (Figure 1A). BALB/c female mice were inoculated with AAV9 twice, 1 week apart, in the quadriceps muscles of alternating hind limbs (“AAV9 directly sensitized dams”) to study the effects of priming of the entire maternal immune system toward AAV antigen. Directly sensitized dams were then mated with male ROSA26^mTmG^ (mTmG)^+/+^ mice to generate pregnancies in directly sensitized dams. To study the isolated effect of maternal humoral immunity, serum was collected from non-pregnant AAV9 directly sensitized dams and adoptively transferred via tail vein injection into a separate cohort of pregnant BALB/c dams on gestational days 15, 16, and 17 (“AAV9 indirectly sensitized dams”). Unsensitized BALB/c dams and BALB/c dams directly sensitized to an alternative AAV serotype (AAV8) served as controls.

AAV9 directly sensitized dams demonstrated significantly elevated concentrations of anti-AAV9 IgG at 28 days after primary inoculation compared with unsensitized and AAV8 directly sensitized dams (Figure 1B), confirming successful induction of a targeted humoral immune response. When AAV9 directly sensitized dams were mated, anti-AAV9 IgG antibodies were efficiently transferred from pregnant dams to their respective mTmG^+/–^ fetuses before birth, with anti-AAV9 concentrations in serum collected from offspring shortly after birth measuring approximately twice as high as those in their respective dams (i.e., fetal/maternal ratio of ~2) (Figure 1C). By contrast, anti-AAV9 IgM antibodies were detected in the dams but not detected in their offspring, demonstrating that vertical transmission of anti-AAV antibodies in mice is IgG selective. Anti-AAV9 IgG binding antibody (BAb) titers were higher in the offspring than in the dams by a factor of 2–4. Anti-AAV9 IgG neutralizing antibody (NAb) titers were higher in the offspring compared with the dams by a factor of 8–16 (Figure 1D).

Having confirmed the induction of anti-AAV antibodies by direct sensitization and characterized their transmission from dam to fetus in murine pregnancy, we next established a model of indirect AAV maternal sensitization and compared anti-AAV immunity following direct versus indirect maternal sensitization. Indirect maternal sensitization by means of serum adoptive transfer successfully transmitted anti-AAV9 IgG antibodies that were efficiently transferred to the fetus, resulting in comparable anti-AAV9 IgG concentrations among the offspring of AAV9 indirectly sensitized and AAV9 directly sensitized dams at birth (Figure 1E). By contrast, IFN-γ ELISPOT analysis demonstrated that maternal anti-AAV9 T cell immunity was present only in AAV9 directly sensitized dams (Figure 1F), confirming that T cells were not transmitted by adoptive transfer. Indirect sensitization therefore resulted in isolated humoral immunity transferred to the fetus.

### Maternal anti-AAV IgG impairs fetal gene editing in a titer-dependent fashion.

We next evaluated the effect of preexisting maternal immunity to AAV on the efficacy of IUGE in the mTmG fluorescent reporter model, in which red fluorescence (mT^+^) is expressed in all cells until deletion of a *loxP*-flanked cassette switches the fluorescence to green (mG^+^). Specifically, AAV9 carrying the SpCas9 transgene and a single-guide RNA (sgRNA) targeting the *loxP* sites (AAV9.SpCas9.sgloxP) was intravenously delivered to gestational day 16 mTmG^+/–^ fetuses in AAV9 directly or indirectly sensitized dams. Gestational day 16 fetuses carried by unsensitized and AAV8 directly sensitized dams served as controls. Two weeks after in utero injection, the livers of unsensitized offspring and AAV8 directly sensitized offspring showed numerous mG^+^ cells by ultraviolet stereomicroscopy and immunofluorescence microscopy, confirming successful gene editing. By contrast, the livers of AAV9 directly sensitized and AAV9 indirectly sensitized offspring showed no mG^+^ cells (Figure 1G), suggesting that anti-AAV9 maternal humoral immunity was sufficient to prevent editing in fetal livers. In order to determine whether the effect of maternal anti-AAV antibodies on fetal gene editing was dose dependent, additional dams were indirectly sensitized to AAV9 with serially diluted (1:10 and 1:100) serum from AAV9 directly sensitized dams, and fetal liver gene editing was quantified by flow cytometry (Figure 1H). Liver editing was equivalent among unsensitized offspring (mean, 2.6%), AAV8 directly sensitized offspring (mean, 2.2%), and AAV9 indirectly sensitized (serum diluted 1:100) offspring (mean, 4.3%). Low-level editing (mean, 0.5%) was observed among AAV9 indirectly sensitized (serum diluted 1:10) offspring, and no editing was observed among AAV9 directly and indirectly sensitized (undiluted serum) offspring. Furthermore, the mean fetal liver editing of each litter was correlated with the anti-AAV9 IgG BAb titer of the respective dam (Figure 1I). Fetal gene editing was absent when maternal titer was greater than 1:25 and was restored when maternal titer was less than 1:25.

### Maternal immune reexposure to AAV affects pregnancy outcomes.

Maternal direct sensitization to AAV9 significantly increased fetal mortality after IUGE in comparison with unsensitized dams, indirectly sensitized dams, and saline-injected controls, with only 4 of 46 (8.7%) injected mTmG^+/–^ fetuses surviving to birth following maternal direct sensitization (Supplemental Figure 1A; supplemental material available online with this article; https://doi.org/10.1172/JCI179848DS1). Stillbirth was the most common outcome for the individual fetus (Supplemental Figure 1B), and total litter loss was the most common outcome for the pregnancy (Supplemental Figure 1C). Maternal serum anti-AAV9 IgM and IgG levels increased after IUGE, consistent with exposure of the maternal immune system to AAV9 as a result of the fetal injections (Supplemental Figure 1D). No differences in maternal T cell frequency, including activated CD4^+^ or CD8^+^ T cells, in the maternal peripheral blood or draining lymph nodes were observed among the groups at 36 hours after IUGE (Supplemental Figure 1, E and F). Additionally, preexisting maternal immunity to AAV9 did not result in increased trafficking of maternal T cells into the fetal blood or fetal liver following IUGE (Supplemental Figure 1G). However, directly sensitized dams did have elevated levels of the proinflammatory cytokines/chemokines IL-6 and CXCL1 at 36 and 96 hours after IUGE. Elevated levels of IL-6 at 36 hours after IUGE were similarly present in the fetuses of directly sensitized dams (Supplemental Figure 2A). Acute inflammation was observed at the basal plate (maternal-fetal interface) of the placenta in aborting and, to a lesser extent, viable fetuses in the AAV9 directly sensitized groups at 36 hours after IUGE, consistent with the cytokine profile (Supplemental Figure 2B). These findings suggested that the mechanism of the increased mortality among fetuses carried by AAV9 directly sensitized dams was reexposure of the primed maternal immune system to AAV9 antigen at the time of IUGE resulting in a systemic inflammatory response affecting the placenta.

### Maternal-fetal transmission of humoral immunity but not T cell immunity to Cas9 endonuclease.

**Figure 2:**
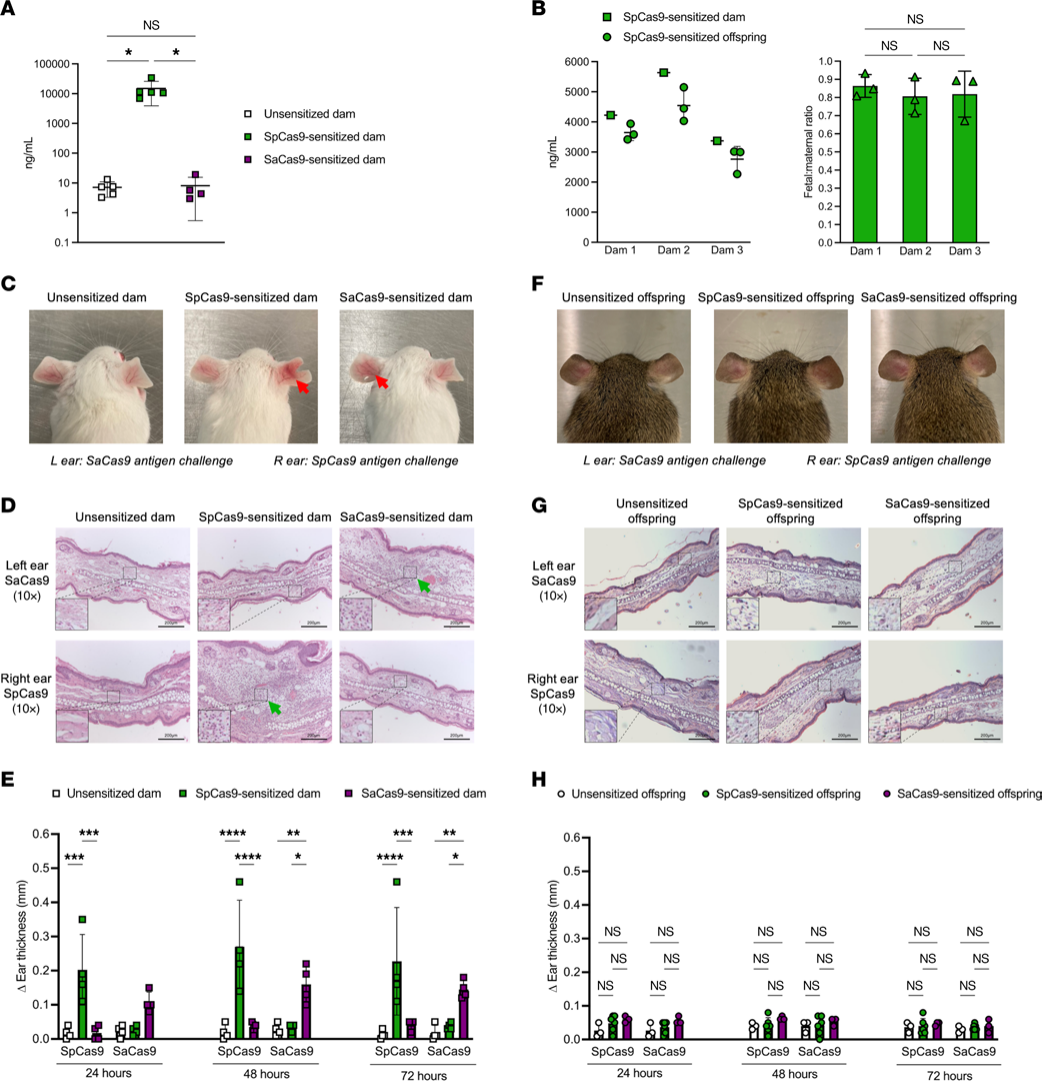
Maternal humoral immunity to Cas9 endonuclease is transferred to the fetus, while maternal T cell immunity to Cas9 endonuclease is not. (**A**) Anti-SpCas9 IgG BAb ELISA. Serum concentrations of anti-SpCas9 IgG BAb were compared among the groups 28 days after primary sensitization using 1-way ANOVA. (**B**) Maternal-fetal transmission of anti-SpCas9 IgG BAb. Shown are the absolute and relative concentrations of anti-SpCas9 IgG BAb among three SpCas9-sensitized dams and their respective newborn fetuses. The mean fetal/maternal ratio for each pregnancy was compared by 1-way ANOVA. (**C**) Anti-Cas9 delayed-type hypersensitivity (DTH) reaction gross histology. Shown are images of the bilateral ears 48 hours after intradermal injection with Cas9 protein. Red arrows point to erythema and swelling consistent with a positive DTH reaction. (**D**) Ear skin microscopic histology. Shown are cross-sectional H&E-stained slides of the ears 72 hours after intradermal injection imaged at ×10 original magnification with ×25 original magnification inset images. Green arrows point to infiltration with small blue lymphocytes and associated edema consistent with a positive DTH reaction. Scale bars: 200 μm. (**E**) Measurement of ear thickness. Ear thickness was measured using microcalipers at 24, 48, and 72 hours after injection and compared among the groups using 2-way ANOVA. Significantly increased thickness was observed in the ears of SpCas9-sensitized and SaCas9-sensitized dams injected with the same Cas9 subtype to which they had been sensitized. (**F**–**H**) Anti-Cas9 DTH reaction among offspring. Unsensitized, SpCas9-sensitized, and SaCas9-sensitized BALB/c dams were mated with mTmG^+/+^ males and allowed to deliver. Pups were fostered and tested at 6 weeks of age. Gross histology (**F**), microscopic histology (**G**), and change in ear thickness compared by 2-way ANOVA (**H**) all demonstrated a negative anti-Cas9 DTH reaction, showing that maternal T cell immunity was not efficiently transferred from dam to offspring. Scale bars: 200 μm. *P < 0.05, **P < 0.01, ***P < 0.001, ****P < 0.0001.

Having demonstrated that preexisting maternal immunity to AAV impaired fetal gene editing in a titer-dependent fashion in the mTmG mouse model, we next evaluated preexisting maternal immunity to Cas9 endonuclease. Immunization of BALB/c females to Cas9 resulted in a Cas9-specific (SpCas9 vs. SaCas9) humoral immune response with elevated anti-SpCas9 IgG antibodies (Figure 2A) that, after mating to mTmG^+/+^ males, were transferred to their mTmG^+/–^ fetuses before birth (Figure 2B). Cas9 immunization also resulted in a Cas9-specific T cell immune response among the dams confirmed by positive delayed-type hypersensitivity (DTH) reaction. Specifically, ears of SpCas9-sensitized BALB/c dams became swollen, erythematous, and indurated after injection with SpCas9 protein (Figure 2C) with histology confirming lymphocytic infiltration characteristic of T cell–mediated inflammation (Figure 2D). This effect was consistent within groups, resulting in significantly increased ear thickness only in response to challenge with the same Cas9 subtype to which the animal had been previously immunized (Figure 2E). In contrast to anti-Cas9 antibodies, anti-Cas9 T cell responses were not observed among the mTmG^+/–^ offspring of SpCas9-sensitized dams. SpCas9-sensitized offspring exhibited negative DTH reactions, demonstrated by absent ear swelling/erythema/induration (Figure 2F), absent lymphocytic infiltration on histology (Figure 2G), and normal ear thickness (Figure 2H) after Cas9 subtype–specific intradermal challenge. The maternal-fetal transmission of T cell immunity, therefore, was inefficient compared with humoral immunity and insufficient to produce a detectable anti-Cas9 T cell response among offspring.

### Maternal Cas9 immunity does not impair fetal gene editing.

**Figure 3:**
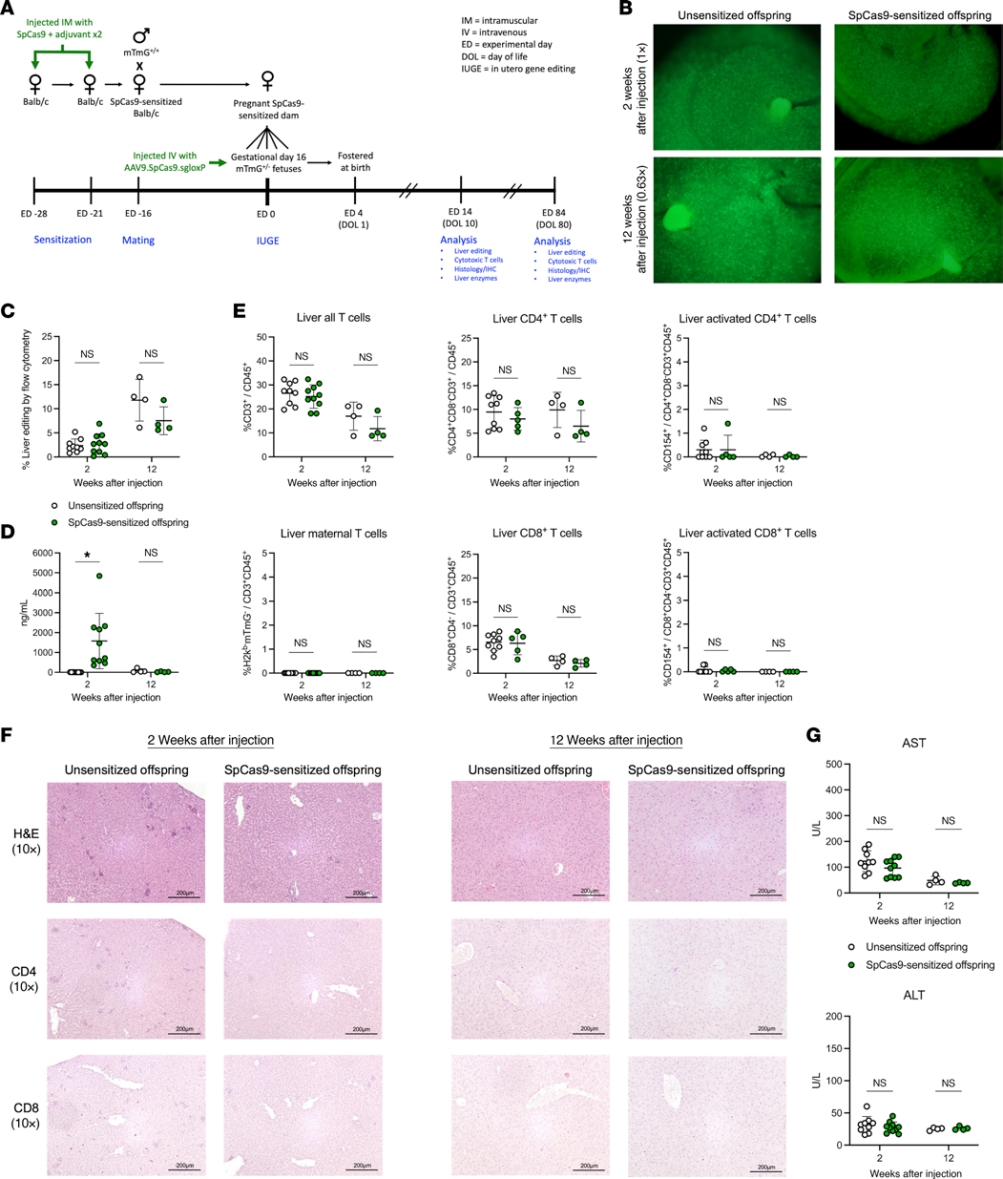
Preexisting maternal immunity to Cas9 endonuclease does not impair fetal liver gene editing. (**A**) Experimental design. (**B**) Stereomicroscopy. Numerous mG^+^ cells were observed in the livers of unsensitized and SpCas9-sensitized offspring at both 2 and 12 weeks after injection. (**C**) Quantification of liver editing by flow cytometry. Shown is percent mG^+^mT^–^/(mG^+^mT^–^ + mG^–^mT^+^) among live CD45^–^TER119^–^CD31^–^EpCAM^–^E-cadherin^+^ offspring hepatocytes compared by 2-tailed *t* test. Comparable editing was observed between the groups in the short and long term. (**D**) Anti-SpCas9 IgG BAbs. SpCas9-sensitized offspring demonstrated increased levels of anti-SpCas9 IgG BAbs on day of life 10 (2 weeks after injection) by 2-tailed *t* test, but these fell to levels comparable to those of unsensitized offspring by 12 weeks after injection. (**E**) T cell analysis. The prevalence of various T cell populations in the liver was assessed by flow cytometry and compared between groups by 2-tailed *t* test. No maternal T cells were detected in livers of SpCas9-sensitized offspring. Cytotoxic T cells were not present at increased frequency in livers of SpCas9-sensitized offspring, nor was a marker of activation (CD154) increased among them. (**F**) Liver histology and IHC. H&E slides were prepared and imaged at ×10 original magnification. Normal hepatocyte morphology without lymphocytic infiltration was observed in both groups in the short and long term. To assess for tissue-infiltrating T cells, IHC for CD4 and CD8 was performed and imaged at ×10 original magnification. Scant CD4^+^ helper T cells and CD8^+^ cytotoxic T cells were detected in equal frequency between the groups. Scale bars: 200 μm. (**G**) Serum transaminases. Serum aspartate transaminase (AST) and alanine transaminase (ALT) were measured 2 and 12 weeks after injection and compared between groups by 2-tailed *t* test. No elevation of AST or ALT was observed among SpCas9-sensitized offspring compared with unsensitized offspring, confirming the absence of hepatocellular injury. *P < 0.05.

Having characterized the vertical transmission of anti-Cas9 humoral and T cell responses in murine pregnancy, we next evaluated whether preexisting maternal immunity to Cas9 impaired the efficacy of IUGE in the mTmG fluorescent reporter model. AAV9.SpCas9.sgloxP was injected intravenously into gestational day 16 mTmG^+/–^ fetuses carried by SpCas9 directly sensitized and unsensitized BALB/c dams. Fetal liver editing, fetal anti-SpCas9 IgG antibodies, T cell infiltration of the fetal liver, and fetal hepatocellular injury were assessed at 2 and 12 weeks after injection (Figure 3A). Numerous mG^+^ cells were visualized on stereomicroscopy within the livers of SpCas9-sensitized and unsensitized offspring at both the short- and the long-term time point (Figure 3B) consistent with successful and sustained liver gene editing. Quantification of editing by flow cytometry demonstrated equivalent editing at both time points with no loss of edited cells (Figure 3C). Anti-SpCas9 IgG was detected at elevated concentrations in offspring of SpCas9-sensitized dams consistent with vertical antibody transmission (Figure 3D). These antibody levels decreased to a low level by 12 weeks after injection, equivalent to offspring from unsensitized dams. Flow cytometry analysis demonstrated equivalent T cell frequency in the liver with no increase in the proportion of CD8^+^ T cells or the expression of CD154, a marker of T cell activation, in offspring of SpCas9-sensitized dams (Figure 3E). Maternal T cells were notably absent in the offspring livers (Figure 3E). No hepatocellular injury was observed on histologic examination of the liver (Figure 3F), and no increase in CD4^+^ or CD8^+^ T cells was detected in the livers of offspring of SpCas9-sensitized dams by IHC at either the 2-week or the 12-week time point. Consistent with this, serum transaminase levels were comparable between the groups (Figure 3G). Finally, survival to birth among fetuses of SpCas9-sensitized dams was comparable to that among offspring from unsensitized dams (58.8% vs. 42.5%, *P* = 0.16) and comparable to survival following PBS injection in fetuses (57.1%, *P* = 0.91). In aggregate, these data demonstrated that preexisting maternal humoral and T cell immunity to Cas9 endonuclease had no detrimental effects on either the efficacy or the safety of IUGE in fluorescent reporter mice.

We next repeated our experiment in unsensitized and SpCas9-sensitized mTmG^+/–^ adult recipients (Supplemental Figure 3A) to confirm that our Cas9 immunization protocol and subsequent gene editing strategy would result in liver-specific inflammation and loss of editing in adult mice consistent with the published literature (14). Loss of editing at 12 weeks after injection was observed by stereomicroscopy (Supplemental Figure 3B) and flow cytometry (Supplemental Figure 3C) among SpCas9-senstized mTmG^+/–^ adults. This was preceded by increased prevalence of CD8^+^ T cells in the liver at 2 weeks after injection and associated with increased expression of the activation marker CD154 among CD8^+^ T cells in the liver at 12 weeks after injection (Supplemental Figure 3D). Consistent with the flow cytometry data, histologic examination of the livers demonstrated increased prevalence of predominantly CD8^+^ T cells in the liver at 2 weeks after injection (Supplemental Figure 3E). Serum liver function tests revealed concurrent transaminitis consistent with hepatocellular injury (Supplemental Figure 3F). These results reproduced the published finding that preexisting Cas9 immunity in adult mice induces a loss of edited cells due to a cytotoxic T cell response, and they confirmed that the absence of detrimental effects on IUGE by preexisting maternal immunity to Cas9 that we observed was not a false negative.

### Maternal immunity to AAV but not Cas9 impairs therapeutic gene editing for an inherited metabolic liver disease.

**Figure 4:**
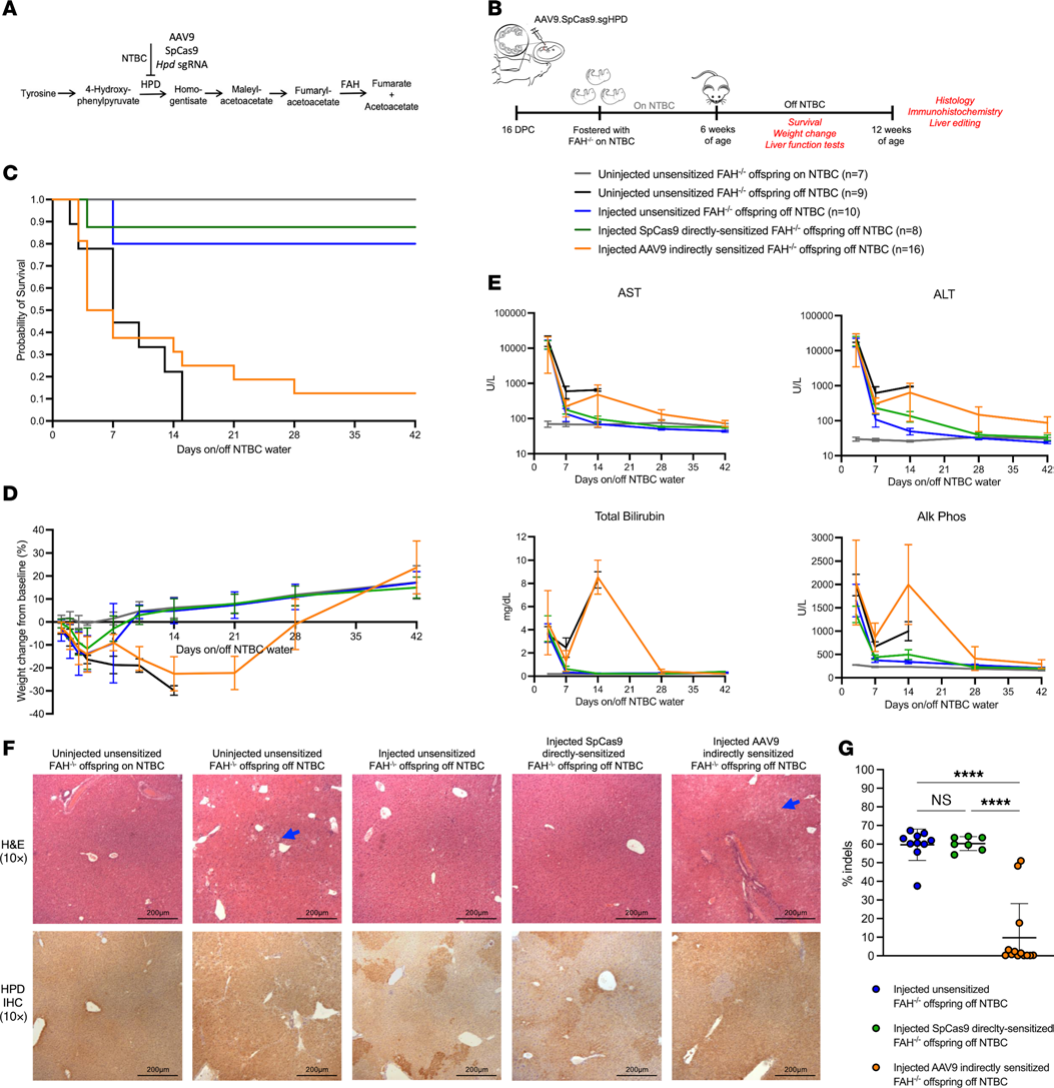
Hereditary tyrosinemia can be cured by AAV9-delivered CRISPR/Cas9 in utero gene editing in the presence of maternal preexisting immunity to Cas9 but not AAV. (**A**) Tyrosine degradation pathway. We designed an sgRNA to induce indels in the *Hpd* gene, thereby replicating the effects of nitisinone (NTBC) through CRISPR/Cas9 gene editing. (**B**) Experimental design. (**C**) Kaplan-Meier survival curve. Injected offspring of unsensitized and SpCas9-sensitized dams demonstrated comparable survival off NTBC by log-rank (Mantel-Cox) test, while significantly decreased survival was observed among injected offspring of AAV9 indirectly sensitized dams. (**D**) Weight change from baseline. Transient weight loss was observed after NTBC withdrawal among injected offspring of unsensitized and SpCas9-sensitized dams with return to baseline after 10 days. Severe weight loss preceded death among injected offspring of AAV9 indirectly sensitized dams (similar to disease controls), and those few that did survive showed prolonged weight loss prior to eventual recovery. (**E**) Liver function testing. Hepatocellular injury was transient among injected offspring of unsensitized and SpCas9-sensitized dams. By contrast, injected offspring of AAV9 indirectly sensitized dams showed prolonged and, in most cases, unresolved hepatocellular injury and cholestatic liver failure. (**F**) Histology. Shown are H&E and IHC for HPD at ×10 original magnification. Characteristic signs of liver injury including hepatocyte ballooning (feathery) degeneration (blue arrows) were observed among injected offspring of AAV9 indirectly sensitized dams off NTBC. Loss of HPD expression by IHC was observed in a patchy pattern among injected offspring of unsensitized and SpCas9 directly sensitized dams. This was notably decreased among injected offspring of AAV9 indirectly sensitized dams. Scale bars: 200 μm. (**G**) Liver editing. Shown is the percentage insertions/deletions (indels) at the *Hpd* locus by next-generation sequencing compared by 1-way ANOVA. Significantly impaired editing was observed among AAV9 indirectly sensitized offspring but not SpCas9 directly sensitized offspring. ****P < 0.0001.

Having demonstrated the effects of preexisting maternal AAV and Cas9 immunity on low-level fetal editing in a fluorescent reporter model, we next evaluated whether the observed trends would persist in a mouse model of an inherited metabolic liver disease wherein edited cells have a survival advantage resulting in high-level editing. *Fah^–/–^* mice, a model of HT1, universally develop hepatitis, failure to thrive, and death in the absence of nitisinone (NTBC), a repurposed herbicide that blocks the activity of 4-hydroxyphenylpyruvate dioxygenase (HPD) in the tyrosine catabolic pathway (Figure 4A). Gestational day 16 *Fah^–/–^* fetuses carried by unsensitized, AAV9 indirectly sensitized, and SpCas9 directly sensitized *Fah^–/–^* dams were intravenously injected with an AAV9 containing the SpCas9 transgene and an *Hpd-*targeting sgRNA that results in the inactivation of the *Hpd* gene (AAV9.SpCas9.sgHPD). At 6 weeks of age, the injected offspring were taken off NTBC and were evaluated for survival, weight loss, liver injury (biochemical and histologic), and liver editing (Figure 4B). Uninjected offspring of unsensitized *Fah^–/–^* dams taken off NTBC served as disease controls, and uninjected offspring of unsensitized *Fah^–/–^* dams maintained on NTBC served as healthy controls.

In utero injection of AAV9.SpCas9.Hpd resulted in 80% survival off NTBC among offspring of unsensitized *Fah^–/–^* dams (Figure 4C). Transient weight loss was observed in the first 4 days off NTBC with recovery to baseline by 10 days and continued weight gain above baseline thereafter (Figure 4D). This was associated with a transient elevation in liver enzymes and hyperbilirubinemia (Figure 4E) consistent with death of unedited hepatocytes with subsequent repopulation of the liver by edited cells. At terminal analysis, liver architecture was normal with decreased expression of HPD by IHC (Figure 4F). Next-generation sequencing of the *Hpd* locus confirmed high-level editing with a mean of 60% insertion and deletion of bases (indels) at the target site (Figure 4G).

Maternal AAV9 indirect sensitization significantly impaired therapeutic fetal gene editing, with only 12.5% of offspring surviving to the study endpoint (*P* < 0.001 for equality of survivor curves). Prolonged weight loss and liver injury were observed among all AAV9 indirectly sensitized offspring, including the 2 survivors. By contrast, offspring of SpCas9-sensitized dams demonstrated equivalent survival (87.5% vs. 80%, *P* = 0.68) and weight change off NTBC (*P* ≥ 0.32 at all time points) compared with injected offspring of unsensitized dams. Equivalent editing by IHC and next-generation sequencing was also observed. These data demonstrated that preexisting maternal humoral immunity to AAV is a persistent barrier to IUGE even in disease models in which edited cells have a marked survival advantage. Conversely, these data showed that preexisting maternal immunity to Cas9 endonuclease remains inconsequential to IUGE.

### Anti-AAV IgG antibodies in human pregnancy; prevalence and efficiency of maternal-fetal transmission at different gestational ages.

**Figure 5:**
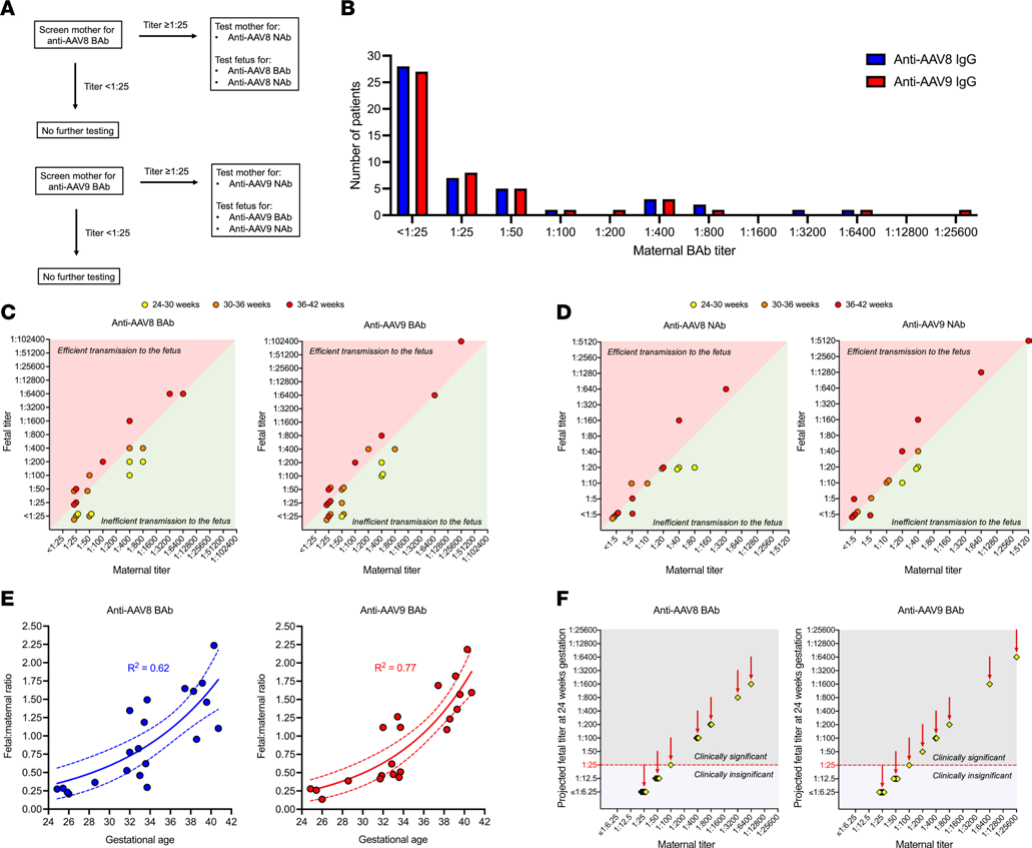
Maternal-fetal anti-AAV antibody transmission efficiency is determined by gestational age in human pregnancy. (**A**) Testing strategy. Maternal serum was first tested for anti-AAV8 and anti-AAV9 IgG BAbs. Titers of 1:25 or higher were considered seropositive and prompted additional testing of both mother and fetus. Titers lower than 1:25 were considered seronegative, and no further testing of either mother or fetus was performed. (**B**) Histogram of maternal BAb titers. Less than half of mothers screened positive for AAV8 and AAV9, with low-level immunity (<1:200) being most common among the seropositive cases. (**C**) Comparison of maternal and fetal BAb titers. Shown are the fetal titer (*y* axis) and maternal titer (*x* axis) for each dyad screening seropositive for anti-AAV IgG. Fetal titers were generally higher than maternal titers at 36–42 weeks gestation, equal to or below maternal titers at 30–36 weeks gestation, and far below maternal titers at 24–30 weeks gestation. (**D**) Comparison of maternal and fetal NAb titers. NAb followed a pattern similar to BAb. Note that hemolysis interferes with luciferase-based assays, resulting in falsely elevated titers. NAb data extracted from hemolyzed samples were therefore excluded. (**E**) Nonlinear regression analysis of fetal/maternal IgG BAb ratio by gestational age. Ratio values greater than 1 indicate efficient antibody transmission to the fetus, and values less than 1 indicate inefficient transmission to the fetus. (**F**) Projected fetal titer at 24 weeks. Using the results of the regression analysis demonstrating a fetal/maternal ratio of 0.25 at 24 weeks, fetal titer at 24 weeks gestation was projected for all seropositive mothers. For 12 of 20 (60%) mothers seropositive for AAV8 and 13 of 21 (62%) mothers seropositive for AAV9, the fetal titer at 24 weeks gestation was projected to be less than 1:25 and therefore clinically insignificant.

Having demonstrated that the effects of preexisting maternal immunity to AAV and Cas9 initially characterized in healthy reporter mice also applied to a model of an inherited metabolic liver disease, we next performed translational studies of preexisting maternal immunity to AAV in humans to determine the efficiency of vertical AAV antibody transmission to the fetus at different gestational ages. We collected serum samples from a total of 48 maternal-offspring dyads. Maternal peripheral blood and neonatal cord blood were collected from routine deliveries in 44 cases (91.7%), and maternal peripheral blood and fetal cord blood were collected from in utero blood transfusions (IUTs) in 4 cases (8.3%). Two (4.2%) pregnancies had twins. Samples were obtained from a broad range of gestational ages: 19 (39.6%) from 36–42 weeks, 19 (39.6%) from 30–36 weeks, and 10 (20.8%) from 24–30 weeks. Testing for anti-AAV8 and anti-AAV9 BAb and NAb was performed as described in Figure 5A with BAb titer ≥ 1:25 considered seropositive.

The overall maternal seropositivity rate was 41.7% for AAV8 and 43.8% for AAV9, with low-level immunity (BAb < 1:200) being most common among the seropositive cases (Figure 5B). One of 4 IUT cases was seropositive (BAb titer 1:400 for both AAV9 and AAV8), and for that case we were able to collect fetal blood twice, once at 25 weeks 3 days and once at 28 weeks 4 days. Both collections were included in the subsequent analysis of vertical transmission efficiency. Among seropositive cases, maternal and fetal BAb titers were compared (Figure 5C). Fetal titers were generally higher than maternal titers at 36–42 weeks gestation, equal to or below maternal titers at 30–36 weeks gestation, and far below maternal titers at 24–30 weeks gestation. A similar trend was observed among seropositive cases for anti-AAV8 and anti-AAV9 NAb titers (Figure 5D). In order to model the effect of gestational age on vertical antibody transmission more precisely, an exact fetal/maternal BAb ratio was calculated for each seropositive pregnancy, and regression analysis was performed (Figure 5E). This demonstrated a strong negative correlation between gestational age and the efficiency of maternal-fetal transmission (*R*^2^ = 0.62 for anti-AAV8 IgG and *R*^2^ = 0.77 for anti-AAV9 IgG), such that at 24 weeks gestation the fetal/maternal antibody ratio was only 0.25. Noting that the target gestational age for a first-in-human trial of IUGE is 18–24 weeks (the age at which fetal umbilical vein injection becomes technically feasible), we next used the predicted fetal/maternal ratio generated by our regression analysis and the known maternal titer to project the fetal titer at 24 weeks gestation for each seropositive pregnancy. In most seropositive cases (12 of 20 [60%] for AAV8 and 13 of 21 [62%] for AAV9), the projected fetal BAb titer fell below the seropositive cutoff of 1:25 (Figure 5F). Of the 48 total pregnancies analyzed, therefore, only 8 (16.7%) would have midgestation fetal titers ≥ 1:25 for AAV8 and only 8 (16.7%) would have midgestation fetal titers ≥ 1:25 for AAV9. Using the less conservative seropositive cutoff of >1:50 used in the onasemnogene abeparvovec trials (3), only 7 of 48 (14.6%) for AAV8 and 6 of 48 (12.5%) for AAV9 would be excluded for antibody levels above the threshold.

## Discussion

Preexisting immunity to the naturally derived components of gene editing technology, including delivery vehicles and bacteria-based editing enzymes, remains a barrier to the broad successful translation of therapeutic gene editing (28, 29). IUGE takes advantage of normal developmental properties of the fetus, including a tolerogenic immune system, to institute a one-and-done therapy before the onset of pathology (30). However, the impact of preexisting immunity when present in the mother has not been previously investigated and must be elucidated before first-in-human clinical trials. Here we have reported the effects of preexisting maternal immunity to AAV vectors and Cas9 endonuclease on liver-directed gene editing in the fetus, demonstrating that maternal humoral immunity to AAV vectors is a titer-dependent barrier to successful IUGE while maternal humoral and T cell immunity to Cas9 endonuclease is not.

In our fluorescent reporter mouse model, preexisting anti-AAV IgG was efficiently transmitted from dam to fetus before birth such that the concentration was higher in the offspring than in their respective dam. This was consistent with both our prior investigation of maternal immunity as a potential barrier to in utero cellular transplantation (24) and physiologic studies elucidating that maternal-fetal antibody transmission in mice, occurring directly via neonatal Fc receptors in the yolk sac splanchnopleure and indirectly through the amniotic fluid via the exocoele, strongly favors the fetus after gestational day 12 (31). While our subsequent finding that preexisting maternal anti-AAV antibodies impaired IUGE in both our fluorescent reporter and metabolic liver disease mouse models suggests that a first-in-human trial of IUGE using AAV vectors would require screening mothers for preexisting AAV antibodies similarly to clinical trials for postnatal gene therapy (3), it is important to consider species-specific differences in maternal-fetal antibody transmission. In humans, maternal-fetal transmission of IgG occurs via the placenta mediated by neonatal Fc receptors expressed on syncytiotrophoblast cells and begins at low levels at 14 weeks gestation (20). Our analysis of human serum collected from maternal-fetal dyads showed that the efficiency of anti-AAV IgG transmission from mother to fetus was highly dependent on the gestational age of the pregnancy, with a 6-fold reduction in the fetal/maternal BAb ratio at 24 weeks gestation (0.25) compared with term (1.75), consistent with prior studies of vertical transmission of pathogen-specific IgG (20, 32, 33). This suggests that the maternal titer we observed to be sufficient to impair gene editing in murine IUGE may be insufficient to impair IUGE in the midgestation human fetus owing to inefficient placental transfer. Similarly, it suggests that the titer sufficient to impair postnatal gene editing in the pediatric or adult patient may, when present in a mother, be insufficient to impair gene editing in her midgestation fetus. As such, the maternal anti-AAV BAb titer used for the exclusion criteria of a IUGE trial could, in theory, be higher than that used for postnatal gene therapy trials, broadening eligibility for enrollment. In our survey of 48 pregnant patients presenting to our centers, for instance, the incidence of maternal titers greater than 1:200 (the maternal titer associated with a projected midgestation fetal titer >1:50) was low (14.6% for AAV8 and 12.5% for AAV9), suggesting that only a small percentage of potential candidates for IUGE would be excluded because of preexisting maternal AAV immunity. This logic, of course, assumes sufficient editing with a one-and-done injection in midgestation and becomes more complex in scenarios in which repeat dosing later in pregnancy is required.

Our observation that preexisting maternal immunity to Cas9 endonuclease does not impair fetal gene editing is a second noteworthy finding. While alternative, less immunogenic delivery vehicles (34) may someday circumvent preexisting AAV immunity in adults, Cas9 remains the backbone of many in vivo editing strategies, including base editing, being investigated in clinical trials (35, 36). If indeed fetal injection circumvents Cas9 immunity in the mother, it would represent a major benefit for the prenatal gene editing strategy. Preexisting maternal Cas9 immunity could induce an anti-Cas9 immune response in the fetus in several ways: (a) direct cytotoxicity from Cas9-specific T cells elicited in the mother (13, 14) and trafficked to the fetus (27); (b) antibody-dependent cellular cytotoxicity initiated by vertically transmitted maternal antibodies and executed by offspring-derived T cells (37); or (c) pregnancy-induced posttranslational antibody modification enabling direct targeting of intracellular antigens (38). Despite confirmed T cell immunity in the dam and efficient vertical transmission of anti-Cas9 IgG to the fetus, no impairment or loss of fetal editing, maternal- or fetal-derived T cell infiltration of the liver, or hepatocellular injury was observed in our reporter model, nor was loss of fetal editing, persistent hepatitis, or decreased survival off NTBC observed in the HT1 mouse model. In contrast, we did observe loss of liver editing, cytotoxic T cell infiltration/activation, and hepatocellular injury when we delivered our vector to SpCas9-sensitized adults, replicating the effect of preexisting Cas9 immunity previously reported in other models of liver-directed and muscle-directed adult gene editing (13, 14). This increased our confidence that the absence of an anti-Cas9 immune response (39) following fetal injection was, in fact, the result of maternal-fetal immune physiology and not a false negative.

Nevertheless, these findings must be viewed with caution given important differences between murine models of IUGE and potential clinical translation. Murine gestation is quite short, lasting only 19–20 days, and as a result fetuses receiving IUGE on gestational day 16 remain in utero for only 72–96 additional hours after the therapy. This contrasts with our paradigm for clinical translation, wherein we would perform fetal intravenous injection at 18–24 weeks gestation, only about halfway through a 40-week pregnancy. It is likely that neoantigen presentation was not robust while the fetus was still in utero in either mouse model, and it is therefore possible that we would observe more maternal T cell trafficking into the fetus in a large-animal model in which a longer period of gestation follows IUGE. For this reason, it will be important to include pregnancies with preexisting maternal Cas9 immunity in large-animal studies of IUGE to confirm the findings in mice reported here.

The differences between murine models and human therapy must also be considered in evaluating the potential effects of preexisting maternal immunity on safety. We observed an increased incidence of stillbirth only among the AAV-injected offspring of AAV9 directly sensitized dams, with all other groups, including AAV9 indirectly sensitized dams and SpCas9 directly sensitized dams, demonstrating survival to birth equal to that observed following fetal saline injection. Based on elegant studies by MacKenzie and colleagues (26, 27, 40), we initially suspected that the increased fetal demise resulted from maternal-fetal trafficking of AAV-specific maternal T cells, which were only present in this group. Our analysis of maternal T cell populations within fetal tissues did not support this conclusion, however, and subsequent experiments demonstrated that the increased incidence of stillbirth more likely resulted from reexposure of the primed maternal immune system to AAV antigen resulting in a systemic maternal inflammatory response characterized by elevated IL-6 and CXCL1 and affecting the placenta. Indeed, both IL-6 and CXCL1 have been implicated in the pathogenesis of adverse pregnancy outcomes, including stillbirth, preterm labor, and chorioamnionitis (41–47), as has maternal infection/reinfection with naturally occurring AAV during pregnancy (48). It is important to note, however, that our murine model of preexisting maternal immunity used repeated immune priming in close proximity to fetal injection, resulting in uniformly high-level immunity. By contrast, preexisting maternal immunity in humans results in heterogeneous, often low-level immunity resulting from remote infection (6). Furthermore, murine IUGE is performed on multiple-gestation pregnancies (large litters) via injection into the vitelline vein, which courses along the amnion in close proximity to the thin-walled uterus, leading to a high probability of injectate contact with maternal tissues due to intraperitoneal leaks and resorption of previously injected fetuses (25). By contrast, human IUGE would be performed similarly to in utero blood transfusions (IUTs) by means of ultrasound-guided injection of singleton fetuses via the umbilical cord (49), which is sequestered within the amniotic cavity away from maternal tissues, and is therefore not expected to result in maternal exposure to the injectate (50). In aggregate, these differences suggest that the mechanism of increased fetal demise elucidated in our study is unlikely to occur in clinical translation. Nevertheless, the finding provides additional impetus beyond impaired efficacy to exclude women presenting with high-level preexisting AAV immunity from IUGE clinical trials.

In summary, preexisting maternal immunity to AAV vectors but not Cas9 endonuclease impairs liver-directed IUGE in murine models, and preexisting anti-AAV antibodies are transmitted inefficiently from mother to fetus in midgestation human pregnancy. Therefore, in utero gene editing may circumvent barriers posed by preexisting immunity to an AAV delivery vehicle or Cas9 endonuclease.

## Methods

### Sex as a biological variable.

Our study examined the immunology of pregnancy, and therefore only female mice were immunized to AAV and Cas9. Transfer of humoral/cellular immunity and associated liver-directed gene editing were studied among male and female offspring, which cannot be differentiated reliably on days of life 0 and 10. Results are therefore reported as a single cohort combining results from both sexes. For the human studies, all maternal blood samples were from biologically female participants. The sexes of the fetuses and newborns from which matched blood samples were obtained included both male and female. The sex of the fetus or newborn was not captured, and thus the data were assessed as an aggregate.

### Fluorescent reporter model.

BALB/cJ (BALB/c, H2k^d+^, catalog 000651) and ROSA26^mTmG^ (mTmG, H2k^b+^, catalog 007576) were purchased from The Jackson Laboratory. The mTmG mouse harbors a constitutively expressed membrane-bound Tomato (mT) flanked by *loxP* sites. Successful cleavage at the *loxP* sites with non-homologous end joining (NHEJ) repair and gene deletion results in constitutive expression of membrane-bound GFP (mG). Using this model, we designed an AAV9 to deliver SpCas9 and a guide RNA to target the *loxP* sites (AAV9.SpCas9.sgloxP) such that editing could be quantified by the expression of mG. Heterozygous offspring of BALB/c females and mTmG homozygous males were injected so that only 1 copy of the mTmG cassette was present (improving discrimination of edited versus unedited cells by flow cytometry) and maternal T cells could be distinguished from fetal T cells by the absence of both mT and H2k^b^ (for T cell trafficking analysis).

### Fah^–/–^ mouse model.

Fah^1R^Tyr^c^/RJ (*Fah*, catalog 018129) mice, a model of HT1, were purchased from The Jackson Laboratory. *Fah* mice were purchased as heterozygotes, subsequently bred to homozygosity (*Fah^–/–^*) in our animal facility, and maintained on nitisinone (NTBC; Yecuris catalog 20-0028) in their drinking water at a concentration of 16.5 mg/L. NTBC blocks the activity of HPD, which is encoded by the *Hpd* gene. *Fah^–/–^* mice universally develop hepatitis, failure to thrive, and death in the absence of NTBC delivered via their mother’s breast milk prior to weaning and in their water supply thereafter. We designed an AAV9 containing the SpCas9 transgene and a guide RNA for induction of indels in the murine *Hpd* gene (AAV9.SpCas9.Hpd). After weaning, injected offspring were maintained on NTBC until 6 weeks of age, at which time NTBC was abruptly withdrawn (i.e., no cycling). Survival, weight change, and liver function tests were serially assessed until 12 weeks of age, at which time survivors were sacrificed for terminal analysis including liver histology, IHC for HPD expression, and next-generation sequencing of the *Hpd* locus in the liver. Uninjected *Fah^–/–^* offspring taken off NTBC and maintained on NTBC served as diseased and healthy controls, respectively. Immunologic responses to antigen and placental biology differ between inbred strains. Injected AAV9 indirectly sensitized *Fah^–/–^* dams were confirmed to have anti-AAV9 IgG titers comparable to those of the injected AAV9 indirectly sensitized BALB/c dams bred to mTmG^+/+^ males to generate mTmG^+/–^ offspring in the fluorescent reporter studies.

### Maternal direct and indirect sensitization.

To induce preexisting maternal immunity to AAV, adult female BALB/c mice were inoculated with AAV9 or AAV8 with a CMV promoter with no transgene (AAV9.CMV.null, Vector Biolabs catalog 7030, or AAV8.CMV.null, Vector Biolabs catalog 7077) at a dose of 10^10^ vg/mouse in 75 μL 5% sorbitol solution without adjuvant. Injection was performed twice, 1 week apart, in the quadriceps muscles of alternating hind limbs (Figure 1A). The resulting mice are referred to as “AAV9 directly sensitized dams” or “AAV8 directly sensitized dams” and were used to study the effects of priming of the entire maternal immune system toward AAV antigen prior to IUGE with AAV vectors. In order to study the isolated effect of maternal humoral immunity, passive immunization was also performed on a separate cohort of so-called “AAV9 indirectly sensitized dams.” Passive immunization of BALB/c or *Fah^–/–^* mice was performed by the adoptive transfer of 200 μL serum via tail vein injection on gestational days 15, 16, and 17. Serum was collected from non-pregnant AAV9 directly sensitized dams 28 days after primary inoculation, pooled among 6–9 donors, and frozen at –80°C until use.

To induce preexisting maternal immunity to Cas9 endonuclease, adult female BALB/c and *Fah^–/–^* mice were inoculated with SpCas9 (EnGen Spy Cas9 NLS, New England Biolabs catalog M0646M) or SaCas9 protein (Engen Sau Cas9, New England Biolabs catalog M0654T) at a dose of 5 μg/mouse in 75 μL 5% sorbitol solution with saponin adjuvant (15 μg/mouse; Quil-A, Invivogen catalog 8047-15-2). Injection was performed twice, 1 week apart, in the quadriceps muscles of alternating hind limbs. As cytotoxic T cells have been shown to be the critical component of the anti-Cas9 immune response to gene editing (13, 14), only direct sensitization to Cas9 endonuclease was studied, and BALB/c mice so immunized are referred to as “SpCas9-senstized dams” or “SaCas9-senstized dams” for brevity. Mice were allocated to treatment and control groups at random.

### Fetal injection.

Fetuses of time-dated pregnant mice were injected on gestational day 16 with 10^14^ vg/kg vector as previously described (51). Briefly, a midline laparotomy was performed under isoflurane anesthesia (3%) to expose the uterine horns. The vitelline vein was injected using a programmable microinjector (IM-300 Microinjector, MicroData Instrument Inc.), a 100 μm beveled glass micropipette, and a dissecting microscope. All fetuses in a litter were injected, with missed injections recorded and excluded from analysis. In order to study the isolated effect of maternal-fetal antibody and T cell transmission during pregnancy and exclude maternal antibodies and T cells transferred after birth via breast milk to newborn pups (25), all litters transplanted before birth were fostered immediately after birth with uninjected dams.

### ELISA.

Total binding antibody (BAb) concentration and titer of anti-AAV9 IgG/IgM were assessed by ELISA at 28 days after primary sensitization. Briefly, AAV9 empty capsids (Vigene Biosciences catalog R2-AAV9-ET) were plated at a concentration of 8.8 × 10^7^ viral particles per well in carbonate buffer (Coating Buffer B, Antibody Pair Buffer Kit, Thermo Fisher Scientific catalog CNB0011) on NUNC Maxisorp flat-bottom plates (Thermo Fisher Scientific catalog 44-2404-21). Samples were tested in duplicate at dilutions ranging from 1:12.5 to 1:10,000. Anti-AAV9 IgG (clone ADK9-1R, Progen catalog 610178S) was used to generate the standard curve against which to calculate/compare concentrations between the groups. Goat anti-mouse IgG Fc cross-adsorbed secondary antibody–HRP (Thermo Fisher catalog 31439) diluted 1:2,500 and goat anti-mouse IgM secondary antibody–HRP (Thermo Fisher catalog PA1-84383) diluted 1:1,000 were used as secondary antibodies. Absorbance at 450 nm (OD_450_) was measured using a Varioskan LUX Multimode Microplate Reader (Thermo Fisher Scientific). The efficiency of vertical transmission of anti-AAV9 IgG was calculated by division of fetal concentration by maternal concentration. No anti-AAV9 IgM was commercially available for use as a common standard, and therefore comparisons of IgM between matched pairs of samples (e.g., offspring versus dam or dam after versus before IUGE) were calculated as a relative value by performing serial dilutions of one sample and using that as a standard curve against which to plot the other at a dilution generating an OD_450_ within standard range. BAb endpoint titer of anti-AAV9 IgG was determined as the highest dilution that produced a mean OD_450_ value 3 times greater than the mean OD_450_ of 6 control wells (no test sample).

Total BAb concentration of anti-SpCas9 IgG was similarly measured by ELISA. SpCas9 (EnGen Spy Cas9 NLS, New England Biolabs catalog M0646M) was plated at a concentration of 0.5 μg/well in carbonate buffer and incubated at 4°C overnight. Mouse anti-SpCas9 IgG (clone 4A1, GeneScript catalog A01935-40) was used to generate the standard curve. Goat anti-mouse IgG Fc cross-adsorbed secondary antibody–HRP diluted 1:5,000 was used as secondary antibody. Samples were tested in duplicate at dilutions ranging from 1:100 to 1:10,000.

### Neutralizing antibody assay.

Anti-AAV9 IgG neutralizing antibody (NAb) assay was performed using HEK293 cells and an AAV vector with LacZ transgene with the assistance of the University of Pennsylvania Gene Therapy Program Immunology Core per published protocol (52). Titer values are the serum dilution at which RLU were reduced 50% in comparison with virus control wells (no test sample). Hemolysis is known to interfere with luciferase-based immune assays (53, 54) and can therefore falsely elevate the titer reported by this assay. Some hemolysis is unavoidable in serum collection from newborn mice, and the potential contribution of hemolysis to the elevated fetal/maternal NAb ratios is noted.

### Anti-AAV IFN-γ ELISPOT assay.

T cell immunity was tested by IFN-γ enzyme-linked immunosorbent spot (ELISPOT) assay with the assistance of the University of Pennsylvania Gene Therapy Program Immunology Core per published protocol (52). T cell immunity was assessed against an AAV9 peptide library composed of 15-mer peptides with 10–amino acid overlap covering the VP1 portion of the AAV9 capsid protein. The peptides are pooled in groups of about 50 peptides, resulting in 3 peptide pools total (pools A, B, and C). Data values are expressed as spot-forming units per million cells for AAV9.

### Stereomicroscopy and immunofluorescence microscopy.

Stereomicroscopy was performed using an Olympus MVX10 (Evident) on freshly isolated livers after washing with cold PBS. For immunofluorescence microscopy, liver wedges were fixed overnight in 4% paraformaldehyde solution (Thermo Fisher Scientific catalog J19943-K2), washed, and serially dehydrated in ascending concentrations of ethanol. Staining was performed using anti-GFP (goat polyclonal, 1:250; Abcam catalog Ab6673), anti-RFP (recognizes mT; rabbit polyclonal, 1:50; Rockland catalog 600-401-379), and DAPI (2 μg/mL; Sigma-Aldrich catalog 32670-5MG-F). Fluorescent images were taken using a Leica DMi8 Thunder microscope (Leica Microsystems).

### Flow cytometry.

Hepatocyte editing in the mTmG model was assessed using flow cytometry. Livers were removed, washed in cold PBS, and finely minced with a razor blade. Enzymatic digestion was performed using a combination of 1.8 mg/mL collagenase I (Worthington catalog LS004196), 27.9 mg/mL dispase (Gibco catalog 17105-041), and 0.11 μg/mL deoxyribonuclease I (Worthington catalog LS002138) for 15 minutes at 37*°*C with an additional 5 minutes of incubation after the addition of 1% 0.5 M EDTA (Invitrogen catalog 15575-038). Cells were then washed, filtered, and resuspended for antibody staining as reported in Supplemental Table 1. Liver editing was calculated as percent mG^+^mT^–^/(mG^+^mT^–^ + mG^–^mT^+^) among DAPI^–^CD45^–^TER119^–^CD31^–^EpCAM^–^E-cadherin^+^ hepatocytes, thereby excluding dead cells (DAPI^+^), leukocytes (CD45^+^), erythrocytes (TER119^+^), endothelial cells (CD31^+^), and cholangiocytes (EpCAM^+^) (55). Analysis of T cell populations including maternal/dam and fetal/offspring cytotoxic T cells was also performed by flow cytometry. Livers and lymph nodes were macerated through a 70 μm filter (Corning catalog 431751) and layered over Ficoll-Paque PLUS (Cytica catalog 17144003) to isolate the low-density mononuclear cells. Antibody staining was performed as reported in Supplemental Table 2. Maternal T cells were differentiated from fetal T cells by the expression of MHC class I and mT (maternal T cells are H2k^b–^mT^–^ while fetal T cells are H2k^b+^mT^+^; see Supplemental Figure 1G). All flow cytometry was performed using a BD FACSAria (BD Biosciences).

### Cytokine analysis.

Blood was collected from dams by retro-orbital venipuncture and from gestational day 17.5 fetuses by decapitation. After clotting at room temperature for 30 minutes, serum was isolated by centrifugation at 10,000*g* for 10 minutes and frozen at –80*°*C until analysis. Cytokines were measured with the assistance of the Children’s Hospital of Philadelphia (CHOP) Translational Core using a V-PLEX Plus Mouse Cytokine 19-Plex Kit (Meso Scale Diagnostics catalog K15255G-1) analyzed with an SQ120 Quickplex (Meso Scale Diagnostics).

### DTH reaction.

In order to assess for T cell immunity to Cas9, we performed a DTH reaction (14) 28 days after primary sensitization. Fifteen micrograms of SpCas9 was injected intradermally into the right ear, and 15 μg SaCas9 was injected intradermally into the left ear. Cas9 was diluted in PBS to a volume of 10 μL and injected using a 30-gauge insulin needle (BD Biosciences catalog 328466). Ears were photographed and measured at 24, 48, and 72 hours after injection using digital microcalipers (Jiavarry catalog 20-F). After 72 hours, ear skin was biopsied, fixed in formalin, and stained with H&E for histologic examination.

### IHC.

Livers were fixed in 10% buffered formalin (Fisher catalog SF100-20) and mounted in paraffin for sectioning. For liver T cell analysis, slides were stained in-house with recombinant anti-CD4 antibody (Abcam catalog ab183685) at a dilution of 1:500 and recombinant anti-CD8α antibody (Abcam catalog ab217344) at a dilution of 1:500. Secondary antibody staining was performed with ImmPRESS HRP Horse Anti-Rabbit IgG Polymer Detection Kit, Peroxidase (Vector Laboratories catalog MP-7401-15). For HPD analysis, slides were stained with the assistance of the CHOP Pathology Core with anti-HPD antibody (rabbit polyclonal, St. John’s Laboratory catalog STJ28588-100) at a dilution of 1:1,000. For all IHC, slides were imaged at ×10 magnification on a Leica DMRBE (Leica Biosystems).

### Liver function testing.

Liver function testing including total bilirubin (Roche catalog 05795397190), alkaline phosphatase (Alk Phos; Roche catalog 03333752190), aspartate aminotransferase (AST; Roche catalog 20764949122), and alanine aminotransferase (ALT; Roche catalog 20764957322) was performed on frozen serum samples with the assistance of the CHOP Translational Core using a Cobas C311 analyzer (Roche Diagnostics).

### Next-generation sequencing.

A standard-sized portion of each liver was used to extract genomic DNA using the DNeasy Blood and Tissue Kit (Qiagen) following the manufacturer’s instructions. Extracted DNA was amplified using primers designed with Primer Blast (NCBI) software and containing the Nextera adaptor sequences: HPD forward, 5′-TCGTCGGCAGCGTCAGATGTGTATAAGAGACAGACAGATCTTCACCAAGCCCAT-3′; HPD reverse, 5′-GTCTCGTGGGCTCGGAGATGTGTATAAGAGACAGGCTGCCTGCGAAATCTTACC-3′. The thermocycler conditions used for all first-round DNA PCRs were: 98°C for 30 seconds, 30 × (98°C for 5 seconds, 60°C for 10 seconds × 30, 72°C for 5 seconds), 72°C for 5 minutes. PCR products were visualized via gel electrophoresis and subsequently purified using a PCR Purification Kit (Qiagen). Next, a secondary bar-coding PCR was performed to add Illumina bar codes (Nextera XT Index Kit V2 Sets A–D) using approximately 15 ng of the first PCR product as a template, and the PCR settings 94°C for 3 minutes, 12 × (94°C for 30 seconds, 60°C for 30 seconds, 68°C for 30 seconds), 68°C for 5 minutes. The resulting secondary PCR products were then purified and normalized. Pooled libraries were then quantified using a Qubit 3.0 Fluorometer (Thermo Fisher Scientific), diluted to 8 pM, denatured, and supplemented with 15% PhiX, and then underwent paired-end sequencing on an Illumina MiSeq System. Sequencing data were analyzed using CRISPResso2 v29 (56).

### Human serum sample collection and analysis.

In order to assess the prevalence of humoral immunity to clinically relevant AAV serotypes among our pregnant patient population as well as assess the effect of gestational age on the efficiency of vertical transmission from mother to fetus, we collected serum samples in 2 clinical contexts. First, we collected maternal peripheral blood and neonatal cord blood from routine deliveries (including term, preterm, and extremely preterm deliveries) at the Hospital of the University of Pennsylvania. Second, we collected maternal peripheral blood and fetal cord blood during IUT performed at the Center for Fetal Diagnosis and Treatment at CHOP. Women aged at least 18 years with singleton or multiple-gestation pregnancies at all gestational ages were enrolled. Exclusion criteria were maternal immunodeficiency/immunosuppression (including AIDS, immunoglobulin deficiency, active steroid use, active chemotherapy) or trauma during this pregnancy with suspected direct mixing of maternal and fetal blood (positive Kleihauer-Betke test and/or requiring Rh_o_(D) immune globulin administration). Whole blood was collected and left at room temperature to clot for 30 minutes before centrifugation at 1,500*g* for 10 minutes. Serum was collected and frozen at –80°C until analysis.

Samples were analyzed according to the algorithm in Figure 5A with maternal BAb titers ≥ 1:25 considered seropositive and prompting additional maternal and fetal testing. BAb and NAb titers were assessed with the assistance of the University of Pennsylvania Gene Therapy Program Immunology Core per published protocol (52). BAb titer values are the reciprocal of the highest dilution that produced a mean OD_450_ value 3 times greater than the assay control. Fetal/maternal ratio was calculated by use of the serial maternal dilutions as a standard curve against which to plot the fetal sample at a set dilution generating an OD_450_ within the range generated by the maternal curve. NAb titers are the reciprocal of the highest dilution at which RLUs were reduced 50% in comparison with virus control wells (no test sample). Blood collection from extremely premature infants and midgestation fetuses is susceptible to hemolysis. Given that hemolysis interferes with luciferase-based assays, resulting in falsely elevated titers (53, 54), NAb data extracted from hemolyzed samples were excluded from the analysis.

### Statistics.

Sample sizes were chosen empirically based on studies of preexisting immunity as a barrier to in utero cellular transplantation and postnatal gene editing (14, 24). Continuous, parametric outcomes were compared among 2 groups using 2-tailed Student’s *t* test and among 3 or more groups using ANOVA with Tukey’s multiple comparisons. One-way ANOVA was used for analyses with a single independent variable (e.g., anti-AAV9 IgG concentrations by group), while 2-way ANOVA was used for analyses with 2 independent variables (e.g., IFN-γ spot-forming units per million cells by group by peptide library or change in ear thickness by group by hours after intradermal injection). Normality was assessed before analysis by 2-tailed *t* test and ANOVA. Binary outcomes including survival to birth were compared by χ^2^ or Fisher’s exact test, as appropriate. The equality of survival curves in the *Fah^–/–^* model was assessed using the log-rank (Mantel-Cox) test. For all tests, a *P* value less than 0.05 was considered significant. Statistical analysis and graphing were performed using Prism version 9.4 (GraphPad). Data are reported as mean ± SD.

### Study approval.

Animals were housed in the Laboratory Animal Facility of the Colket Translational Research Building at CHOP. Murine experimental protocols were approved by the CHOP IACUC and followed the guidelines set forth in the NIH’s *Guide for the Care and Use of Laboratory Animals*, 8th edition (National Academies Press, 2011). Protocols for human serum collection, storage, and analysis were reviewed and approved by the Institutional Review Boards of the Hospital of the University of Pennsylvania (849821) and CHOP (20-017423). Written informed consent was obtained from study participants.

### Data availability.

All study data can be accessed in the Supporting Data Values file.

## Author contributions

JSR designed the research study, conducted the experiments, acquired the data, analyzed the data, and wrote the manuscript. VLL, CLB, AMD, NJK, AD, and PM conducted the experiments and acquired the data. MEDP and MGA analyzed the data. LL and RJ acquired the data. LD and CPT collected the samples and analyzed the data. PWZ designed the research study, conducted the experiments, and edited the manuscript. WHP designed the research study, analyzed the data, and edited the manuscript.

## Supplementary Material

Supplemental data

Supporting data values

## Version 1 06/17/2024

Electronic publication
