# Supplementary material for: Preexisting maternal immunity to AAV but not Cas9 impairs in utero gene editing in mice
Source: J Clin Invest. 2024 May 9;134(12):e179848. doi: 10.1172/JCI179848 (PMC11178531; doi:10.1172/JCI179848)
Supplement: Supplemental data [file jci-134-179848-s036.pdf]

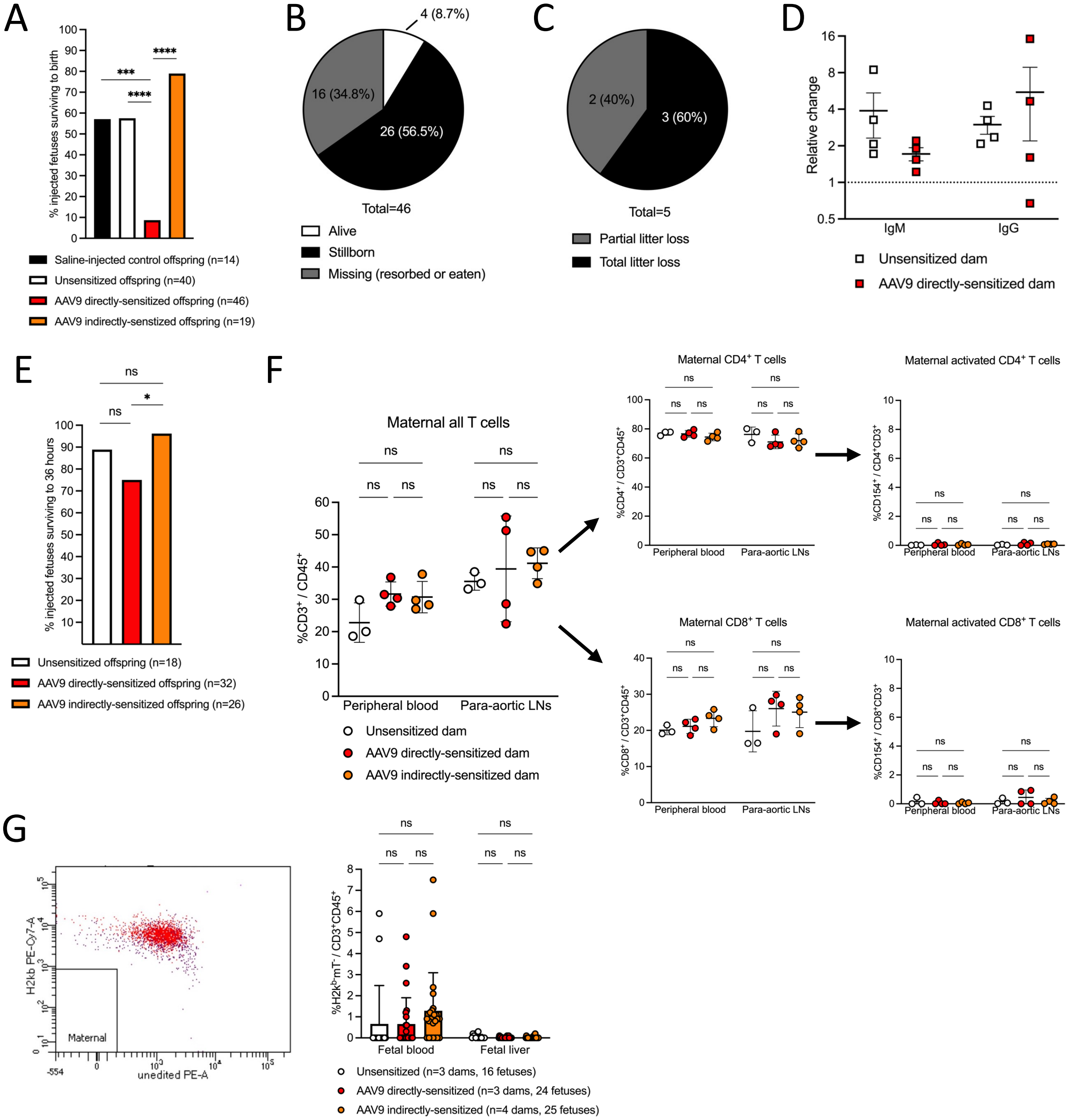

Supplemental Figure 1: IUGE results in re-exposure of the primed maternal immune system to AAV resulting in a systemic inflammatory response detrimental to fetal survival. (**A**) Fetal survival to birth. Shown is the percentage of injected fetuses surviving to birth in each group. Percent survival was compared among the groups using the χ^2 test of proportions. (**B**) Granular fetal outcomes among AAV9 directly-sensitized offspring. Stillbirth was the most common outcome of fetal injection in this group. (**C**) Pregnancy outcomes among AAV9 directly-sensitized dams. In most cases, total loss of the litter was observed. The data presented in panels B and C suggest a process affecting the mother and the pregnancy as a whole rather than a process occurring on the level of the individual fetus (which would more likely present as frequent in utero demise [i.e., more missing/resorbed fetuses] and partial litter loss). (**D**) A comparison of maternal anti-AAV9 IgM and IgG antibodies after IUGE versus before IUGE. Relative change was calculated by dividing the post-injection concentration by the pre-injection concentration. Among unsensitized dams, IgM and to a lesser extent IgG increased after IUGE, suggesting exposure of the maternal immune system to AAV as a result of fetal injection. Among AAV9 directly-sensitized dams, IgG and to a lesser extent IgM increased after IUGE, suggesting re-exposure of the maternal immune system to AAV as a result of fetal injection. (**E**) Short-term survival. Percent survival at 36 hours post-injection was compared among the groups using the χ^2 test of proportions. Most fetuses carried by AAV9 directly-sensitized dams were still viable at this time point and therefore could be analyzed. (**F**) Maternal T cell analysis. Shown is the prevalence of T cell subtypes in the peripheral blood and para-aortic lymph nodes (LN), which represent the draining nodal basin of the uterus. Comparison among the groups was performed using two-way ANOVA. (**G**) Maternal-fetal T cell trafficking. Shown are the gating strategy and percentage of T cells in the fetal blood and fetal liver that are maternal in origin. Maternal T cells are $H2k^{b}mT^{-}$ while fetal T cells are $H2k^{b+}mT^{+}$. No increase in the prevalence of maternal T cells was observed in fetuses carried by AAV9 directly-sensitized dams by two-way ANOVA. Together with panel F, these data show that in AAV9 directly-sensitized dams, maternal T cells were not being activated or trafficking in increased numbers into the fetus as a result of IUGE, suggesting that the maternal T cell response was not likely the cause of the increased fetal mortality observed in this group. *p<0.05, **p<0.01, ***p<0.001, ****p<0.0001.

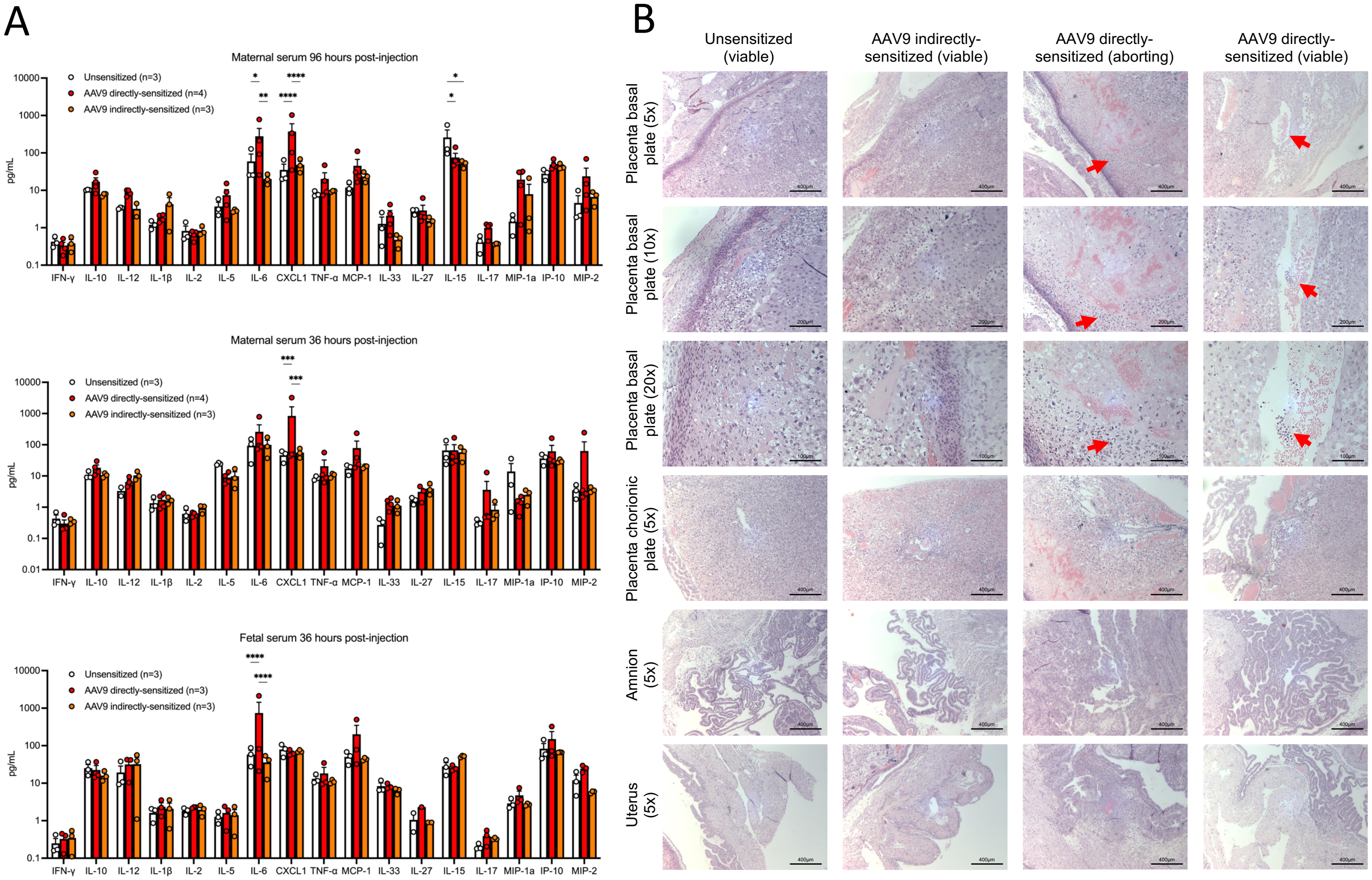

Supplemental Figure 2: IUGE results in re-exposure of the primed maternal immune system to AAV resulting in a systemic inflammatory response detrimental to fetal survival (continued). (**A**) Cytokine analysis. After IUGE, the maternal and fetal serum were analyzed for elevated markers of systemic inflammation by a multiplex cytokine assay and compared among the groups by two-way ANOVA. AAV9 directly-sensitized dams were observed to have increased levels of CXCL1 and IL-6, and IL-6 was similarly elevated in their fetuses. IL-6 and CXCL1 have been implicated in the pathogenesis of adverse pregnancy outcomes. Unsensitized dams showed elevated serum IL-15 at 96 hours post-injection. IL-15 is associated with acute viral infection, and its elevation in the only group without pre-existing immunity to AAV was consistent with the finding from Supplemental Figure 1D that IUGE in mice results in exposure of the maternal immune system to AAV after fetal injection. Bars represent the mean + standard error of the mean for each group. *p<0.05, **p<0.01, ***p<0.001, ****p<0.0001. (**B**) Histology. The placenta (basal and chorionic plates), amnion, and uterus were examined for microscopic features of inflammation 36 hours post-injection. Neutrophil infiltration (red arrows) was observed at the basal plate of the placenta in the AAV9 directly-sensitized group, most pronounced among early fetal deaths but also detectable to a less extent among still-viable fetuses. This was consistent with the elevated CXCL1 observed on the cytokine analysis in panel A. Notably, lymphocytic infiltration of the fetal side of the placenta, uterus, or amnion was not noted in the AAV9 directly-sensitized group, consistent with the results of the flow cytometry analysis of the maternal blood, maternal draining lymph nodes, fetal blood, and and fetal liver at this early time point after injection (see Supplemental Figure 1 F-G).

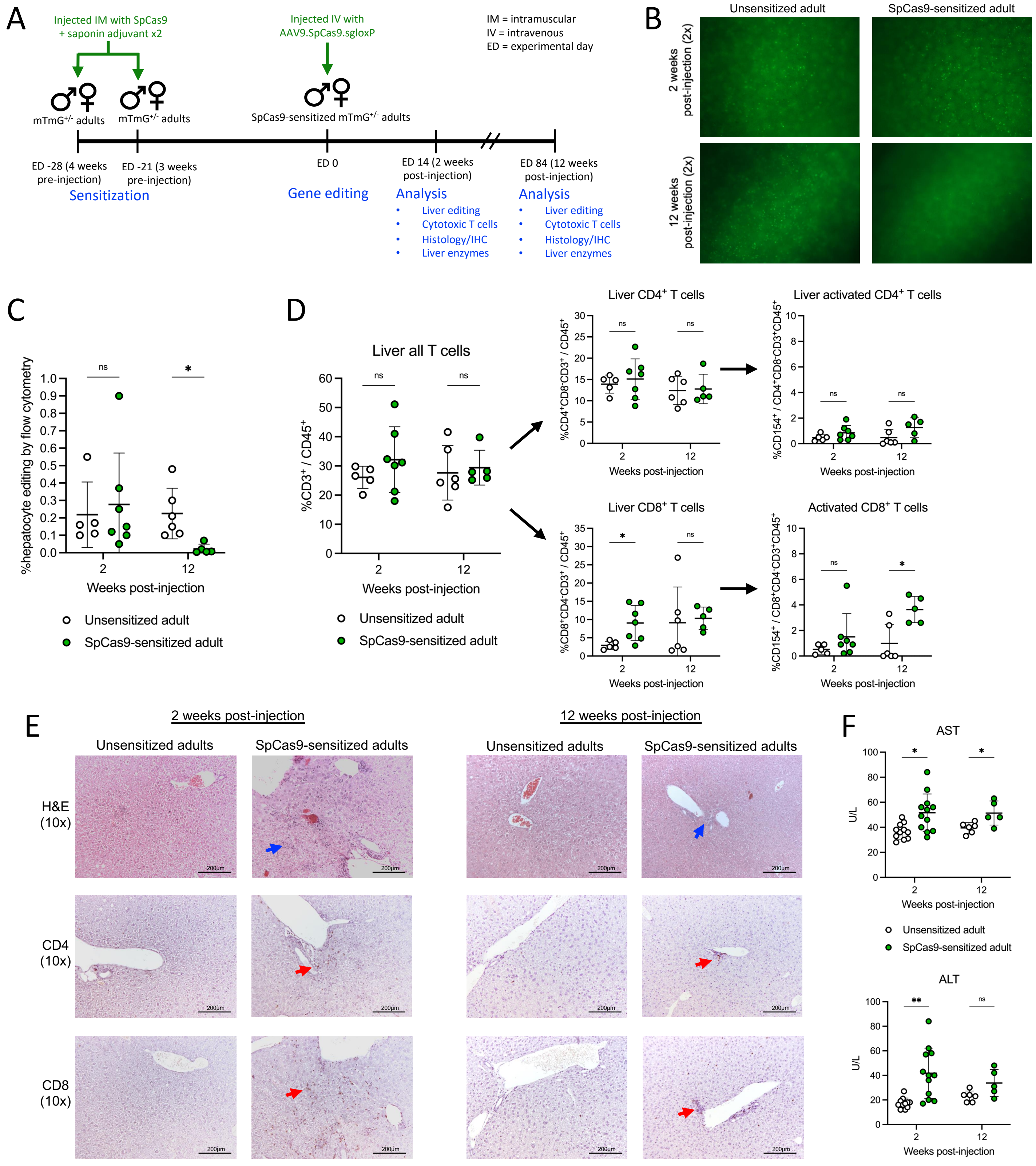

Supplemental Figure 3: Pre-existing immunity to Cas9 endonuclease impairs gene editing in adults. (**A**) Experimental design. mTmG$^{+/-}$ male and female adults were sensitized to SpCas9 by intramuscular (IM) injection of 5μg SpCas9 protein with 15μg saponin adjuvant twice, one week apart. Twenty-eight days after primary sensitization, these SpCas9-sensitized adults and unsensitized adults were injected intravascularly (IV) via the tail vein with AAV9.SpCas9.sgloxP at a dose of 3.75x10^{13}vg/kg. Two and twelve weeks after injection, animals were assessed for liver editing and for cytotoxic T cell liver infiltration with associated hepatocellular injury. (**B**) Stereomicroscopy. Similar numbers of bright green (mG$^+$) cells were observed in the livers of unsensitized and SpCas9-sensitized adults at 2 weeks post-injection consistent with successful gene editing. However, at 12 weeks post-injection almost no mG$^+$ cells were observed in the livers of SpCas9-sensitized adults, suggesting a loss of edited cells. (**C**) Quantification of liver editing by flow cytometry. Shown is %mG$^+$mT$^-$ / (mG$^+$mT$^-$ + mG$^-$mT$^+$) among live CD45$^-$TER119$^-$CD31$^-$Ep-CAM$^-$E-Cadherin$^+$ hepatocytes. Comparable editing by two-tailed t test was observed at 2 weeks post-injection, but at 12 weeks post-injection editing was significantly reduced to zero or near-zero among SpCas9-sensitized adults. (**D**) T cell analysis. Increased prevalence of cytotoxic T cells was observed among SpCas9-sensitized adults at 2 weeks post injection, and increased expression of a marker of T cell activation (CD154) was noted among these cytotoxic T cells at 12 weeks post-injection. (**E**) Histology and IHC. To assess for hepatocellular injury, hematoxylin and eosin (H&E) slides were prepared and imaged at 10x magnification. Lymphocytic infiltration (blue arrows) was observed among SpCas9-Senstized offspring, most notably at 2 weeks post-injection. IHC imaged at 10x magnification demonstrated increased numbers of CD4$^+$ helper T cells and CD8$^+$ cytotoxic T cells (red arrows) infiltrating the livers of SpCas9-sensitized adults only. This was most prominent at 2 weeks post-injection, although lingering T cells were noted at 12 weeks post-injection. These findings were consistent with the flow cytometry data in panel D and together confirm cytotoxic infiltration of the gene-edited liver among SpCas9-sensitized adults. (**F**) Serum transaminases. To assess for hepatocellular injury resulting from cytotoxic T cell infiltration, serum aspartate transaminase (AST) and alanine transaminase (ALT) were measured 2 and 12 weeks post-injection and compared by two-tailed t test. AST and ALT were elevated in SpCas9-sensitized adults at 2 weeks post-injection with some persistent elevation in AST noted at 12 weeks post-injection. These data supporting hepatocellular injury are consistent with the kinetics of T cell infiltration and loss of edited cells demonstrated above. In aggregate, the effects of pre-existing immunity to Cas9 in adults contrasts to the persistent editing, lack of cytotoxic T cell liver infiltration, and lack of hepatocellular injury observed among the offspring of SpCas9-sensitized dams, demonstrating an immunologic advantage for the fetal gene editing approach compared to the adult. *$p<0.05$, **$p<0.01$, ***$p<0.001$, ****$p<0.0001$.

Supplemental Table 1: Flow protocol for liver editing analysis. Staining was performed in a volume of 100µL for 25 minutes at 4ºC. After washing, cells were resuspended in 200µL for DAPI staining and analysis. Fc blockade was not used. Liver editing was calculated as %$mG^{+}mT^{-}$ / ($mG^{+}mT^{-}$ + $mG^{-}mT^{+}$) among live $CD45^{-}TER119^{-}CD31^{-}$Ep-$CAM^{-}$E-$cadherin^{+}$ hepatocytes. Gating for mG was set by fluorescence minus one using hepatocytes from uninjected $mTmG^{+/-}$ controls.

Target	Fluorophore	Clone	Manufacturer	Cat# / Ref#	Dilution
TER119	Alexa Fluor700	TER-119	Biolegend	116220	1:200
CD45	PE-eFluor610	30-F11	eBioscience	61-0451-82	1:100
CD31	PE/Cy7	MEC13.3	Biolegend	102524	1:200
CD324 (E-Cadherin)	PerCP-eFluor710	DECMA-1	eBioscience	46-3249-82	1:100
CD326 (Ep-CAM)	APC/Cy7	G8.8	Biolegend	118218	1:100
LGR5	APC	DA04-10E8.9	Miltenyi Biotec	130-111-202	1:50
Immediately before analysis:					
Live/dead	DAPI	n/a	Miltenyi Biotec	130-111-570	1:100

Supplemental Table 2: Flow protocol for cytotoxic T cell analysis (peripheral blood, lymph nodes, and liver). Staining was performed in a volume of 100µL for 25 minutes at 4ºC. Fc blockade was not used.

Target	Fluorophore	Clone	Manufacturer	Cat# / Ref#	Dilution
CD45	PerCP/Cy5.5	30-F11	Biolegend	103132	1:100
$H2k^{b}$	PE/Cy7	AF6-88.5.5.3	eBioscience	25-5958-82	1:50
CD3	BV421	17A2	Biolegend	100228	1:100
CD4	APC/Cy7	RM4-5	Biolegend	100526	1:50
CD8	PE-eFluor610	53-6.7	eBioscience	61-0081-82	1:50
CD154	APC	MR1	eBioscience	17-1541-82	1:100
